# Intraoperative Magnetic Resonance Imaging Complications Leading to Remote Intracerebral Hemorrhagic Infarction Following a Glioblastoma Resection: A Case Report and Lessons for Clinical Practice

**DOI:** 10.7759/cureus.90554

**Published:** 2025-08-20

**Authors:** Narushi Sugii, Masahide Matsuda, Eiichi Ishikawa

**Affiliations:** 1 Department of Neurosurgery, Institute of Medicine, University of Tsukuba, Tsukuba, JPN

**Keywords:** complication, glioblastoma, intraoperative mri, remote hemorrhagic infarction, venous infarction

## Abstract

Glioblastoma is the most aggressive primary brain tumor, requiring a multidisciplinary strategy with an emphasis on “maximal safe resection” to improve outcomes. Intraoperative magnetic resonance imaging (iMRI) enables real-time assessment of tumors and helps preserve eloquent brain areas. While generally safe, rare complications may arise. We report a rare case of remote hemorrhagic infarction after glioblastoma resection using iMRI, possibly linked to mechanical compression from intraoperative gauze. A 50-year-old male patient with progressive aphasia had a maximal diameter of 55 mm contrast-enhancing lesion in the left medial temporal lobe, consistent with glioblastoma. Resection with iMRI was uneventful, and imaging confirmed total removal without ischemia or hemorrhage. Before iMRI, gauze was inserted into the epidural space, and the skin was temporarily closed. Postoperatively, the patient developed new aphasia and right hemiparesis. Emergency imaging 12 hours after surgery showed a hemorrhagic infarction in the ipsilateral precentral and inferior frontal gyri, remote from the surgical site. Retrospective review of iMRI indicated significant brain compression and venous dilation, suggesting impaired venous return during the 30-minute iMRI scan led to infarction from venous congestion or reperfusion injury. To our knowledge, this is the first report implicating iMRI-related brain compression in remote venous infarction, underscoring the need for cautious intraoperative brain handling to prevent such complications.

## Introduction

Glioblastoma is a highly malignant brain tumor with a poor prognosis, requiring multidisciplinary treatment and emphasizing "maximal safe resection" during surgery [1,2]. In neurological surgery, intraoperative magnetic resonance imaging (iMRI) is a powerful tool for maximizing tumor resection. Potential risks associated with intraoperative MRI include burns, accidents caused by ferromagnetic metal instruments, and an increased risk of infection due to prolonged surgery time for imaging; however, performing iMRI does not significantly increase these risks [3,4].

Ischemic complications following brain tumor surgery are relatively common, with a maximum incidence of approximately 64% when asymptomatic cases are included [5]. On the other hand, remote cerebral infarction and/or hemorrhage occurring at a site distant from the surgical manipulation is very rare, accounting for approximately 1.1% to 1.8% and 0.2% to 1.9% of the cases, respectively [6-9]. Excluding case reports, there are no articles specifically focused on remote infarctions occurring in the cerebrum, but the mechanisms of remote infarctions are speculated to include delayed vasospasm and obstructed venous return due to intra- and postoperative cerebrospinal fluid overdrainage and brain shifts [10-12]. To the best of our research, however, there have been no reports of venous infarction as a complication of iMRI procedures resulting in remote hemorrhagic infarction.

Herein, we report a rare case of remote intracerebral hemorrhagic infarction after glioblastoma resection using iMRI, which may have resulted from a unique iMRI-associated mechanism. We obtained informed consent for publication and anonymized the patient's clinical information in compliance with the Declaration of Helsinki and Japan’s Personal Information Protection Act.

## Case presentation

Preoperative findings

**Figure 1:**
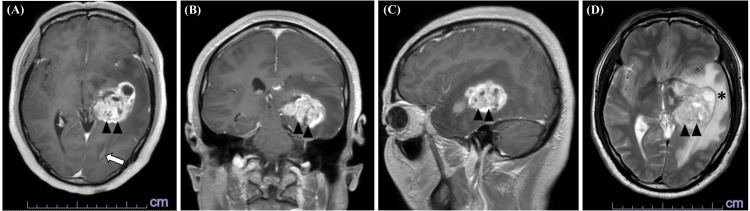
Preoperative MRI findings Initial brain MRI demonstrated a ring-shaped enhanced mass lesion involving the left medial temporal lobe (arrowheads) extending to the wall of the lateral ventricle (arrow) on post-contrast T1WIs of axial (A), coronal (B), and sagittal (C) sequences. The tumor exhibited perifocal edema on T2WI (D, asterisk). MRI: magnetic resonance imaging; WI: weighted image

A 50-year-old right-handed man without any medical history presented to our hospital with progressive aphasia (primarily manifesting as difficulties in reading and writing kanji). An MRI of the head revealed a large mass (maximum diameter: 55 mm) in the left medial temporal lobe (Figure 1). The lesion exhibited ring-shaped contrast enhancement and extended to the wall of the lateral ventricular triangle, suggesting subependymal extension. Glioblastoma was suspected, and a maximal safe resection (excluding the subependymal extension area) was planned using iMRI.

Intraoperative findings

**Figure 2:**
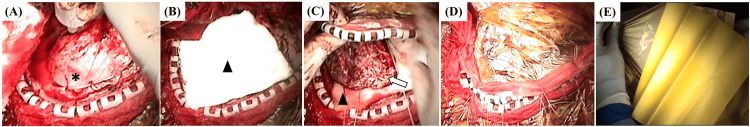
Preparation for intraoperative MRI Before performing intraoperative MRI following tumor removal, the dura mater was closed (A, asterisk), a towel gauze instead of a bone flap was placed (B, arrowheads), the temporal muscle was repositioned (C, arrow), and the skin was temporarily closed (D). The wound was covered with an iodine-impregnated surgical drape (E). MRI: magnetic resonance imaging

During the frontotemporal craniotomy, we divided the deep and superficial layers of the temporalis muscle, with the superficial transverse temporal vein (STTV) being cauterized and dissected at that time. The extraction was completed without any issues or motor-evoked potential (MEP) decline, after which an iMRI scan was performed. To prepare for the iMRI scan at our institution, we placed a towel gauze in the epidural space instead of a bone flap (intended to reduce the number of handling times of the patient's bone flap), roughly sutured the skin, and sealed the wound with an iodine-impregnated surgical drape (Figure 2).

**Figure 3:**
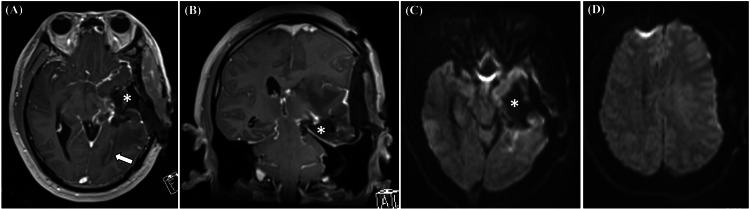
Intraoperative MRI findings Intraoperative brain MRI demonstrated gross total resection of the enhanced tumor (asterisks), except for the subependymal extension of the posterior horn of the lateral ventricle (arrow) on post-contrast T1WIs of the axial (A) and coronal (B) sequences. No obvious bleeding or ischemic complications were observed on DWI (C and D). T1WI: T1-weighted image; DWI: diffusion-weighted image; MRI: magnetic resonance imaging

iMRI confirmed total resection of the target area without apparent ischemia or hemorrhage (Figure 3); therefore, we decided not to perform additional resection and discontinued MEP monitoring following iMRI. The skull and skin were then closed. Intracranial pressure was not monitored during the operation, but no signs of increased intracranial pressure (e.g., brain swelling) were observed upon dural closure.

Postoperative course

**Figure 4:**
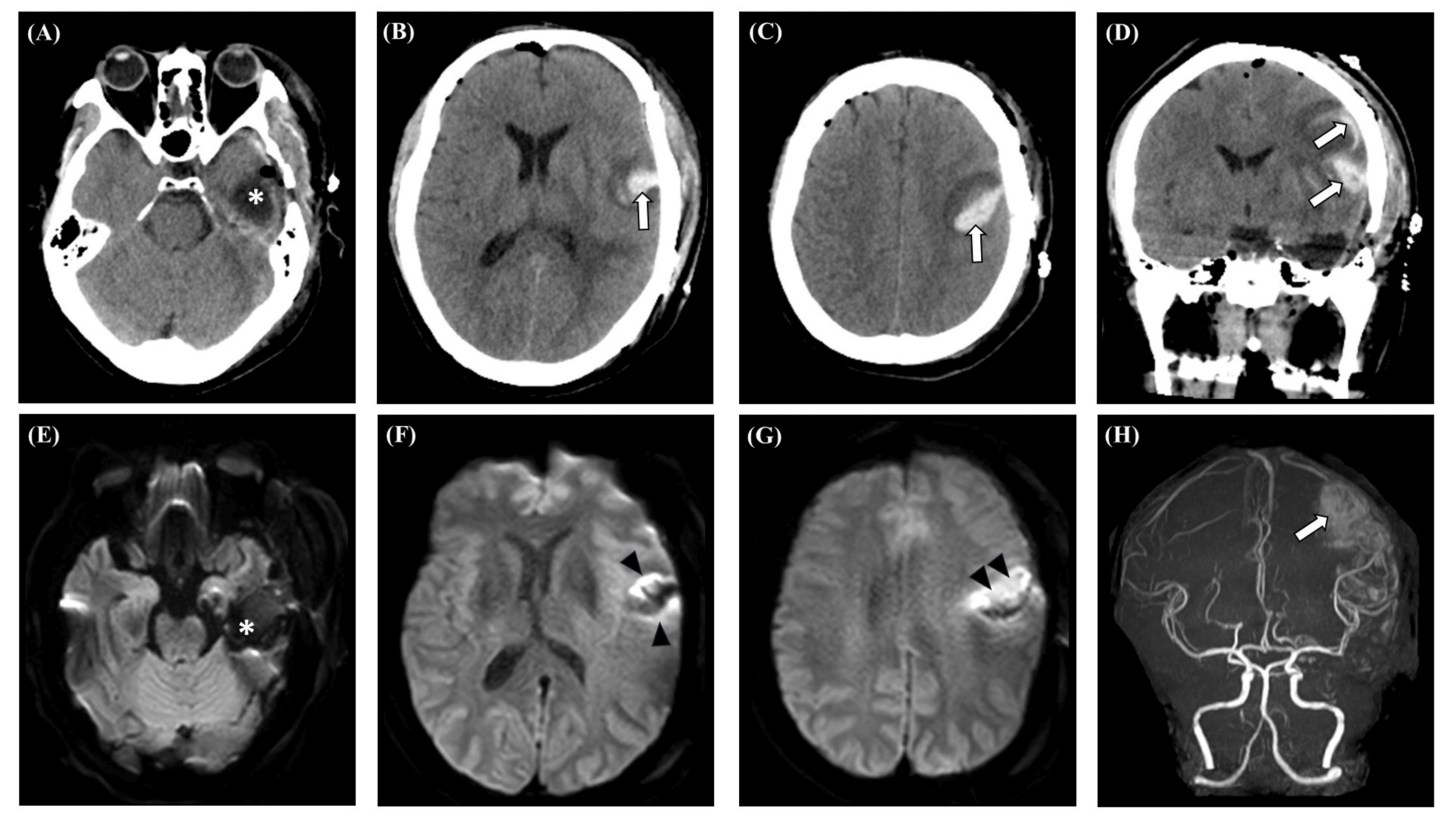
Postoperative imaging findings Postoperative emergent CT of axial (A–C) and coronal (D) images revealed intracerebral hemorrhage around the left precentral gyrus and inferior frontal gyrus (arrows), accompanied by surrounding low-density areas. The asterisks indicate the surgical cavity. Brain MRI performed 12 hours after surgery demonstrated heterogeneous diffusion-restricted areas (arrowheads) at a site distant from the extraction site (asterisks) on diffusion-weighted imaging (E–G), indicating remote hemorrhagic infarction. MRA of the frontal view revealed the presence of major arteries (H). CT: computed tomography; MRA: magnetic resonance angiography; MRI: magnetic resonance imaging

After awakening from general anesthesia, the patient exhibited worsening aphasia and right hemiparesis of the upper limb and face. Since the resection surgery of the dominant temporal lobe could exacerbate the aphasia and the iMRI revealed no abnormalities, we judged the symptoms to be transient and followed up with the patient. However, the following morning (12 hours after surgery), the symptoms did not improve. Emergency computed tomography (CT) and MRI revealed no abnormalities around the tumor resection site; however, an intracerebral hemorrhagic infarction, primarily involving the ipsilateral precentral gyrus and inferior frontal gyrus, which were not surgically manipulated, was observed (Figure 4). Magnetic resonance angiography demonstrated the patency of major arteries, and the extent and characteristics of the lesion suggested a mechanism involving venous occlusion.

**Figure 5:**
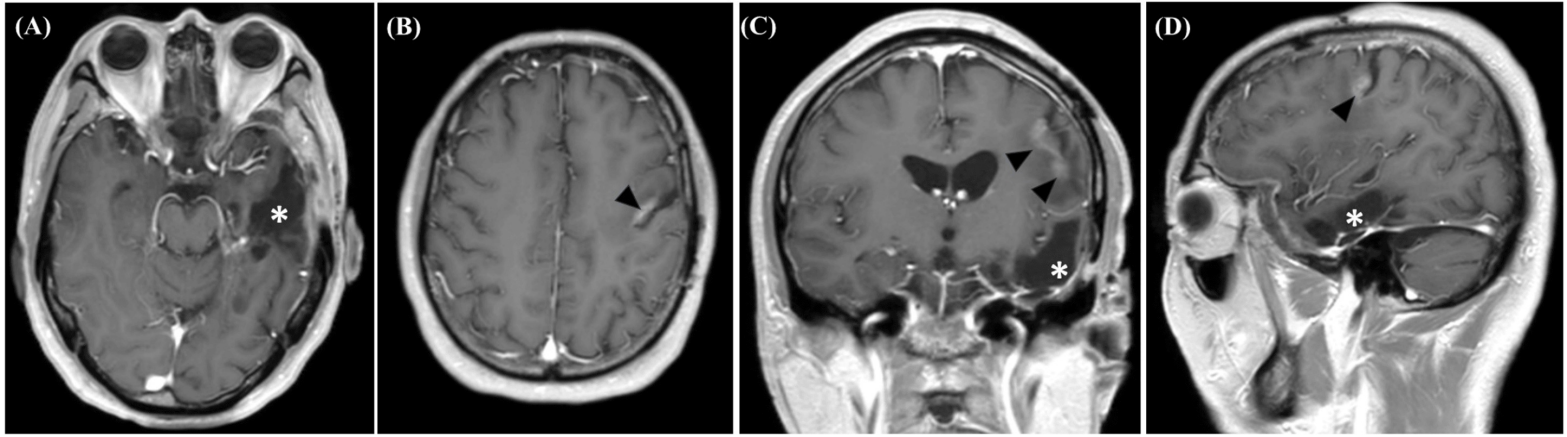
Follow-up MRI findings Brain MRI one year after surgery demonstrated no signs of tumor recurrence but changes following remote hemorrhagic infarction (arrowheads) on post-contrast T1WIs of axial (A and B), coronal (C), and sagittal (D) sequences. The asterisks indicate the surgical cavity. MRI: magnetic resonance imaging; WI: weighted image

Conservative management was implemented for the hemorrhagic infarction, and no aggravation of the condition was observed (Figure 5).

The pathological diagnosis of the tumor was glioblastoma, IDH-wildtype, and the patient underwent chemoradiation therapy. The symptoms gradually improved; however, the patient did not fully recover. The patient was discharged home with a Karnofsky performance status of 60.

Review of iMRI findings

**Figure 6:**
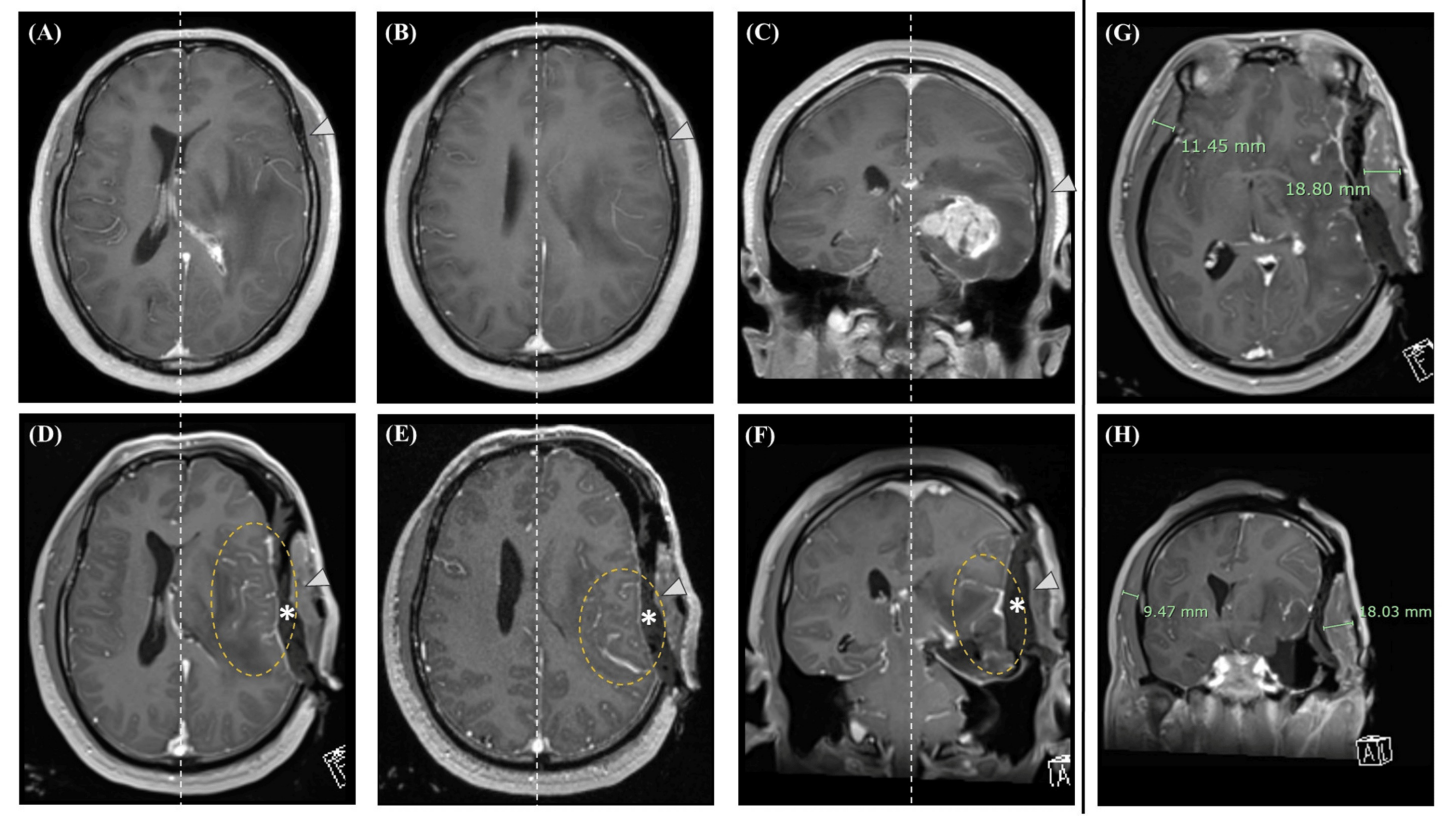
Comparison of pre- and intraoperative MRI findings Compared to the preoperative MRIs (upper column, A–C), intraoperative brain MRIs (lower column, D–F) demonstrated that the left cerebrum was compressed and deformed by the temporal muscle (arrowheads) and the inserted gauze (asterisks). The left cerebral veins were more dilated than those on the contralateral side (dotted circles). Vertical dotted lines indicate midlines. The maximum thicknesses of the temporal muscles on both sides are displayed in the axial (G) and coronal (H) sections. MRI: magnetic resonance imaging

Upon review of the recorded surgical video, no identifiable venous damage was observed that could account for the condition. Then, subsequent review of the iMRI revealed marked brain compression and deformation, attributed to the mass effect of the inserted gauze and the swollen temporal muscle (Figure 6). Moreover, the cerebral veins on the surgical side appeared dilated compared to those on the contralateral side (maximal diameters were 2.4 vs 1.5 mm) and the ipsilateral preoperative findings.

**Figure 7:**
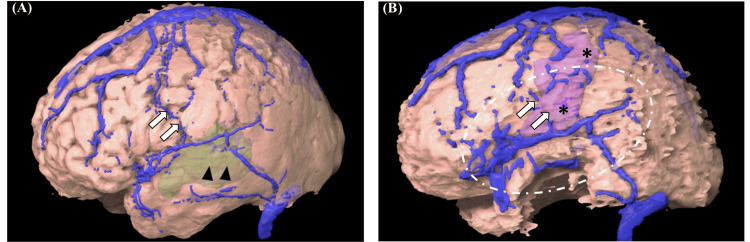
Three-dimensional images of the brain Three-dimensional images created from pre- (A) and intraoperative (B) MRI data are presented. Arrowheads and asterisks indicate enhanced tumors (translucent) and hemorrhagic infarctions (translucent), respectively. The central veins that drain the precentral gyrus near the Sylvian fissure (arrows) were difficult to visualize under compression (dotted circle area) on intraoperative MRI (B). MRI: magnetic resonance imaging

We speculated that mechanical compression caused widespread obstruction of the venous return during the approximately 30-minute iMRI (Figure 7) and that remote hemorrhagic infarction had occurred after the resumption of surgery (i.e., reperfusion of affected vessels) before recovery from general anesthesia.

## Discussion

We encountered a case of glioblastoma where remote intracerebral hemorrhagic infarction occurred after tumor resection using iMRI. Although the exact mechanism is not fully understood, the phenomenon is generally attributed to disturbances in venous circulation. In the present case, during iMRI, the towel gauze placed along with the swollen temporal muscle severely compressed the brain, causing expansion of the cerebral veins, especially on the cranial side of the extraction cavity (Figures 6, 7). This suggests that significant impairment of venous return developed in this region, resulting in hemorrhagic infarction at a site remote from the surgical field. Given the absence of MEP decline before iMRI and the lack of evidence of hemorrhagic infarction on iMRI, it was presumed that the event occurred between the completion of iMRI and the patient’s recovery from general anesthesia.

The primary question in this case was whether the approximately 30-minute duration of iMRI was sufficient to induce venous hemorrhagic infarction. In arterial occlusion, cerebral infarction occurs even with blood flow interruption lasting only 30 minutes. Additionally, hemorrhagic conversion is significantly more likely if reperfusion occurs following an occlusion lasting longer than two hours [13,14]. In cases involving venous mechanisms, although limited data exist regarding the time required for cerebral infarction to occur, experimental studies in cats have demonstrated that intracerebral hemorrhagic infarction occurs within six to 12 hours following extensive occlusion of cortical veins along with the superior sagittal sinus [15]. In addition, hemorrhagic transformation in venous infarction may be caused not only by reperfusion, as in arterial infarction, but also by vascular rupture due to increased venous pressure and blood stasis [16]. The cerebral veins lack venous valves and are vulnerable to sudden increases in venous pressure, leading to hemorrhagic infarctions [17]. In actual clinical practice, the time required for venous infarction to occur is considered to be shorter for hemorrhagic infarction than for non-hemorrhagic infarction [11,18], with one-third of patients with remote cerebellar hemorrhage exhibiting symptoms immediately after surgery [19]. Furthermore, hemorrhagic infarction developed during the 12-hour interval between the conclusion of the iMRI and the initial postoperative imaging study, rather than the 30-minute duration of iMRI imaging. Considering the aforementioned factors, even a short timeframe of 30 minutes can potentially result in hemorrhagic infarction due to impaired venous return.

The following factors are generally involved in the occurrence and severity of venous infarction:(1) degree of venous compression (complete or partial occlusion), (2) presence of collateral blood flow (whether other vessels can compensate for the occlusion), (3) location and type of the affected vein, and (4) patient's general condition (dehydration, abnormalities in the coagulation and fibrinolysis systems, etc.) [16,18,20]. When venous injury occurred during intracranial lesion resection surgery, hemorrhagic infarction developed postoperatively in 37.6% of the cases. In particular, injuries to the central vein, vein of Labbe, or internal cerebral vein inevitably led to hemorrhagic infarction, resulting in severe and irreversible neurological deficits [18]. In this case, although complete occlusion of the vein may not have occurred, significant compression deformation centered around the superficial middle cerebral vein (SMCV) was observed (Figures 6D-6F, 7), resulting in venous failure without compensatory blood flow in the inferior part of the central vein that drained into the SMCV. Additionally, patients undergoing brain tumor surgery frequently exhibit abnormalities in their coagulation and fibrinolysis systems, which may contribute to the development of hemorrhagic infarctions [21].

iMRI has become indispensable for achieving maximal safe resection in glioma surgery, and its use has generally been reported not to increase associated risks [3,4]. However, acknowledging that various complications, such as those related to the procedures employed during iMRI, may occur in individual cases is important. Moreover, in this case, the absence of abnormal findings on iMRI contributed to the delayed timing of postoperative imaging studies. From this perspective, case reports detailing these rare complications are important. When a frontotemporal craniotomy is performed, the temporal muscle undergoes swelling, resulting in an over 20% increase in volume compared with that on the contralateral side [22]. In this case, the temporalis muscle showed a marked swelling of more than 60% compared to the contralateral side (Figures 6G and 6H), which may be exacerbated by the cauterization and dissection of veins within the temporalis muscle (STTV) during craniotomy. In any case, it is a fact that there was significant swelling of the temporal muscle in this case, which is considered to have caused intense pressure on the brain. As a countermeasure for the future, we must pay close attention to the thickness of the temporal muscle and the inserted gauze, and the insertion of gauze should be tailored for each case; in cases where swelling of the temporal muscle is prominent, it might be better to avoid inserting gauze altogether.

## Conclusions

We encountered a rare case of remote hemorrhagic infarction after glioblastoma resection, which was attributed to venous return obstruction caused by excessive brain compression during iMRI. Even though iMRI is a cornerstone of modern glioma surgery, there may be pitfalls in the imaging procedures. It is essential to recognize that the temporal muscle can become swollen during surgery and to take utmost care with the thickness of the inserted gauze, adjusting it for each case. Although rare, in cases of significant temporal muscle swelling, omitting the insertion of gauze may be considered.
